# Traditional Chinese Medicine in Cancer Care: A Review of Case Series Published in the Chinese Literature

**DOI:** 10.1155/2012/751046

**Published:** 2012-05-31

**Authors:** Guoyan Yang, Xun Li, Xiaoli Li, Lu Wang, Jia Li, Xue Song, Jizhong Chen, Yu Guo, Xiaoxuan Sun, Shana Wang, Zhiqi Zhang, Xiaoyun Zhou, Jianping Liu

**Affiliations:** ^1^Centre for Evidence-Based Chinese Medicine, Beijing University of Chinese Medicine, Beijing 100029, China; ^2^School of Humanities, Beijing University of Chinese Medicine, Beijing 100029, China; ^3^School of Preclinical Medicine, Beijing University of Chinese Medicine, Beijing 100029, China; ^4^School of Acupuncture and Moxibustion, Beijing University of Chinese Medicine, Beijing 100029, China; ^5^National Research Center in Complementary and Alternative Medicine (NAFKAM), University of Tromsø, 9037 Tromsø, Norway

## Abstract

Traditional Chinese medicine (TCM) has been widely used in cancer in China. Case series report a series of cases exposed to a certain intervention. To understand the current situation of case series of TCM for cancer, we performed this review. We included case series of cancer patients treated with TCM therapy. Electronic searches were conducted in four main Chinese databases until February 2011. A total of 1,217 reports of case series (92,945 patients) were included. The top five types of cancer were lung cancer, liver cancer, stomach cancer, leukemia, and esophageal cancer. Leukopenia and hiccup treated by TCM were the most common adverse reactions after surgery or induced by chemo/radiotherapy. More than half of the patients were treated with TCM therapies alone. The application of herbal medicines especially formula based on syndrome differentiation was highly prevalent, and the typical administration route was oral usage. 1,182 reports were published in a structured format. The quantity of TCM case series for cancer treatment is substantial. Further studies should focus on the most common types of cancer and the most frequently applied TCM therapies. We presented a recommendation from the methodological point of view for the format of reporting.

## 1. Introduction

Cancer is a leading cause of death worldwide. According to the World Health Organization, it has accounted for 7.6 million deaths (around 13% of all deaths) in 2008, and deaths from cancer worldwide are projected to continue to rise to over 11 million in 2030 [1].

Conventional treatment for cancer, such as surgery and chemo/radio therapy, aims at curing the disease or prolonging life while improving the patient's quality of life (QOL) [1]. However, it is acknowledged that most cancer patients suffer from both the disease itself and symptoms induced by conventional treatment. 

Traditional Chinese medicine (TCM) is a holistic system of medicine including herbal medicine, acupuncture and moxibustion, *tuina*, dietary therapy, and* qigong*. TCM has unique theories of the diagnosis and treatment, which is mostly the syndrome differentiation and prescription of herbal formula. A systematic review of case reports showed rich information of case reports about TCM therapies for cancer and suggested potential benefit [2].

Case series, a classical type of observational study, retrospective or prospective, report and examine the medical results of a series of cases exposed to a certain intervention (prevention or treatment) [3]. According to Oxford grading of evidence, case series are graded level 4, which is generally considered as low strength-level evidence [4]. However, all or none of the case series (“met when all patients died before the Rx (intervention) became available, but some now survive on it, or when some patients died before the Rx became available, but none now die on it”) are graded up to 1c of all 5 levels [5].

There have been a considerable number of case series concerning TCM for cancer published in Chinese journals, yet no systematic summary has been done. Without analyzing these publications, we will not detect any possible valuable information from the considerable amount of evidence. Therefore, this review aims to systematically identify, collect, and summarize case series on TCM in the treatment of cancer published in Chinese journals to provide an overview of current evidence of TCM for cancer treatment. Through this review, we hope to find potentials for further studies and to make recommendations for further TCM case series report from the methodology point of view.

## 2. Material and Methods

### 2.1. Source of Literature and Search Strategy

 Two authors (G. Yang and X. Li) searched the major Chinese electronic databases, including China National Knowledge Infrastructure (CNKI) (1911–February 2011), Chinese Scientific Journal Database (VIP) (1989– February 2011), Chinese BioMedical Literature Database (CBM) (1978– February 2011), and Wanfang Database (Wanfang) (1994– February 2011).

The searching terms used included “*zhong_yi*” (Chinese medicine), “*zhong_yao*” (Chinese herbs), “*zhong_yi_yao*” (traditional Chinese medicine), “*zhong_cheng_yao*” (Chinese patent medicine), “*zhong_cao_yao*” (Chinese herbal medicine), “*zhen_ci*” (acupuncture), “*zhen_jiu*” (acupuncture and moxibustion), “*zhong_xi_yi_jie_he*” (integrated traditional Chinese and western medicine), “*an_mo*” (massage), “*tui_na*” (*Tuina*), “qi_gong” (*Qigong*), “*gua_sha*” (skin scraping therapy), “*ba_guan*” (cupping), “*xue_wei*” (acupuncture point), “*min_zu_yao*” (ethnomedicine), “*min_jian*” (folk), “*ai*” (cancer), “*liu*” (tumor), “*e_xing*” (malignant), “*e_xing_zhong_liu*” (malignant tumor), “*bai_xue_bing*” (leukemia), “*gu_sui*” (bone marrow) and “*lin_ba*” (lymph). No language restriction was applied.

### 2.2. Inclusion Criteria

1. Types of study: case series published in Chinese journals.
2. Types of participants: patients with cancer, including malignant tumor and malignant hematologic disease, or patients in precancerous condition.
3. Types of interventions: Chinese medicine (herbal medicine, patent medicine, acupuncture, moxibustion, *tuina, *dietary therapy, *qigong* and other folk therapies used in China). Chinese medicine integrated with conventional medicine was also included if detailed data of TCM in cancer care were available.
4. Types of outcome measures: survival, relapse or metastasis, complication, symptoms, quality of life, adverse reactions induced by surgery or chemo/radio therapy, or safety.

### 2.3. Literature Selection

 Three authors (G. Yang, X. Li, and L. Wang) screened titles for the eligibility and relevance independently, and full-text versions of the relevant papers were retrieved and reviewed for selection of case series according to the inclusion and exclusion criteria. If there was any uncertainty during the study selection, a fourth author (J. Liu) was consulted.

### 2.4. Data Extraction and Analysis

 A structured data extraction form was designed. It consisted of the following sections.

1. Basic Bibliometric Information. It included full citation and year of publication.
2. Type(s) of Cancer and Related Condition. In reports on cancer, cancer names were extracted and classified into ten categories: musculoskeletal (including bone marrow), dermatological, neurological, endocrine (including breast), hematologic and lymphatic, respiratory, digestive (including liver, gallbladder and pancreas), urinary and reproductive systems, head and neck cancer, and others. In reports on precancerous conditions, symptoms induced by conventional therapies, and other cancer-related conditions, the names were also extracted.
3. Diagnostic Criteria. It was examined whether the three items reported: diagnostic criteria, gold diagnostic standard (pathological diagnosis), and syndrome differentiation (*bianzheng*) based on TCM theory.
4. Type(s) of Intervention. TCM therapy was at first place classified into TCM alone and TCM therapy integrated with conventional therapy. Then, TCM therapy was further classified into herbal medicine, acupoint stimulation, dietary therapy, *tuina*, and *qigong*. Herbal medicine was further classified into formula based on syndrome differentiation (common types of formula in textbook [6]), self-prescribed formula (prescribed by practitioners), Chinese patent medicine, preparation used in specific hospital, injection extracted from herbs, and single-herb preparation. Acupoint stimulation was further classified into manual acupuncture, moxibustion, ear-acupuncture, cupping, electroacupuncture, and acupuncture point injection (injection with western medicine was defined as integrated therapy). We examined whether there was a detailed description of treatment regimens, administration, dosage, and course of the TCM therapy.
5. Outcome Measures. Data of outcome were extracted including survival, relapse and/or metastasis, symptom (including cancer pain and tumor size), quality of life, adverse reaction after surgery or induced by chemo/radio therapy, and safety. For quality of life, we examined whether a standardized measurement was reported. We also examined whether the result was reported as composite outcome with defined grades, which combined more than one outcome criterion in each grade. We also examined whether the author(s) recommended for clinical application.
6. Structure of Reporting. We examined whether the study reported in a structured manner, concerning objective, material and methods (participants, treatment regimens, and outcome measures), results, discussion, and/or conclusion in different sections.

Data were extracted by ten authors and then verified by one author (G. Yang). Any discrepancies were discussed and consensus was reached with involvement of the other authors. Data were presented as counts, percentage, or frequency (calculated by number of publications and by patients respectively due to a wide range of patients number involved in case series).

## 3. Results

### 3.1. Bibliometric Information

**Figure 1:**
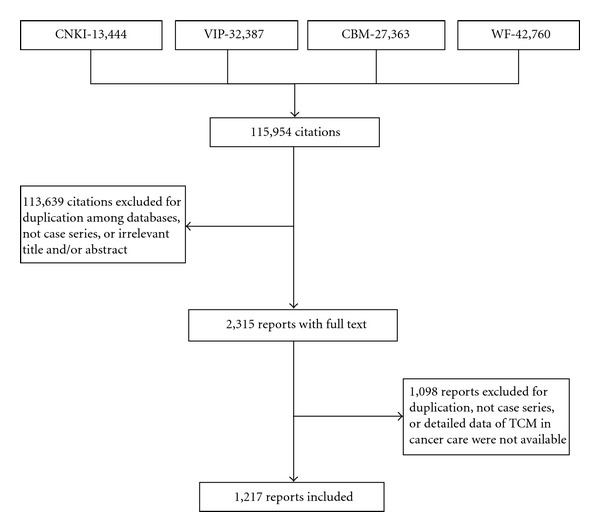
Flowchart of literature search and study selection.

A total of 115,954 citations were identified and screened by two authors (G. Yang and X. Li) based on the searching of the four electronic databases, and 113,639 citations were excluded due to duplication, irrelevant title, and/or abstract. Full texts of the remaining 2,315 citations were retrieved and evaluated according to the inclusion and exclusion criteria, and 1,098 citations were excluded. In total, 1,217 reports of case series involving 92,945 cancer patients were included in this review (Figure 1).

**Figure 2:**
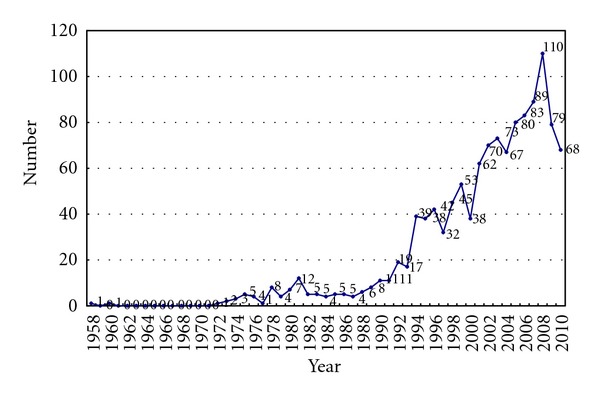
Publications number of case series on TCM for cancer in Chinese journals.

Among the retrieved reports from 1958 to 2011, only 2 [7, 8] were published before 1969. However, the number of publications concerning cancer patients per year appeared to increase from 1972 with the peak of 110 reports in 2008 (no publications were found in 1959 and in 1961–1971) (Figure 2). The number of patients involved in the 1,217 reports varied from 5 to 18,963 patients per study [9, 10]. 1086 (89.24%) reports involved less than 100 patients.

### 3.2. Types of Cancer and Cancer-Related Conditions

 Out of the 1,217 reports of case series, 652 reported the treatment of cancer, 16 reported the treatment of precancerosis (8 for chronic atrophic gastritis and 6 for myelodysplastic syndrome, mainly for the prevention of stomach cancer and leukemia), and 549 reported the treatment of adverse reactions after surgery or induced by chemo/radio therapy and complications, of which cancer pain, leucopenia, effusion in thoracic cavity or abdominal cavity, and hiccup were most frequently reported (Table 1).

Among all the 652 reports covering ten categories of cancer, a total of 27 different types of cancer were included. The top three categories were digestive cancer, respiratory cancer and hematologic/lymphatic cancer (Table 2). 

**Figure 3:**
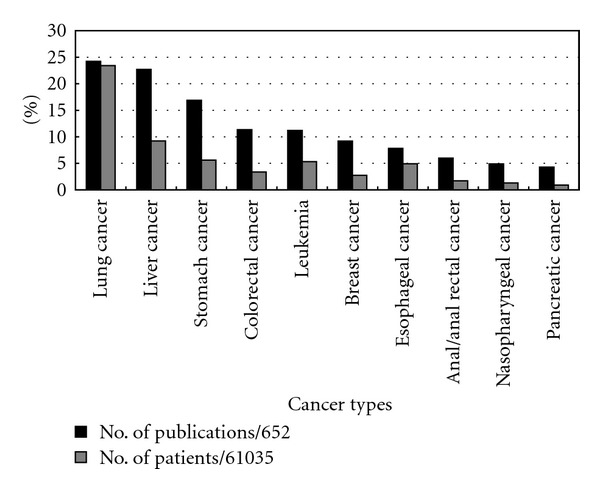
Top ten types of cancer treated by TCM in case series published in Chinese journals.

Lung cancer, liver cancer, and stomach cancer were the top three cancer types according to frequency both calculated by number of publication, and patients. Leukemia was the 5th and 4th most frequent cancer calculated by number of publications and by patients, respectively (Figure 3).

### 3.3. Diagnostic Criteria

In the 652 case series reports on cancer treated by TCM, 542 (83.13%) described the diagnostic criteria, 478 (73.31%) described the gold standard (pathological diagnosis), 407 (62.42%) described the cancer stage, and 364 (55.83%) described TCM syndrome differentiation. 

In the remaining 565 reports on precancerous diseases, symptoms induced by chemo/radio therapies or other cancer-related conditions, 396 (70.09%) described the diagnostic criteria, 245 (43.36%) described the gold standard (pathological criteria), 135 (23.89%) described the cancer stages, and 247 (43.72%) described TCM syndrome differentiation.

### 3.4. Types of Intervention

 Among the 1,217 reports, 678 (55.71%) with 61,529 (66.20%) cancer patients reported the application of TCM therapy alone and 539 (44.29%) with 31,690 (34.10%) cancer patients reported the integration of TCM and conventional medicine.

A total of 4912 (5.28%) patients have received more than one type of TCM therapy in 73 (6.00%) reports. Out of the 1,217 reports, herbal medicine and acupoint stimulation were most frequently applied. The top three applications of herbal medicine were formula based on syndrome differentiation, self-prescribed formula, and Chinese patent medicine. Types of acupoint stimulation mainly involved manual acupuncture, acupuncture point injection, and electroacupuncture (Table 3).

Among the 652 reports for the treatment of cancer, herbal medicine was mostly reported in 640 (98.16%) reports involving 59,918 (98.17%) patients, while *tuina* was not used. 

For the administration of herbal medicine in the 1,217 reports, oral medication applied in 912 (74.94%) reports with 77,080 (82.93%) patients, external application in 163 (13.39%) with 8,802 (9.47%), and injection in 158 (12.98%) with 7,559 (8.13%) were most frequently reported types. Oral medication was most frequently applied for formula based on syndrome differentiation. The administration of injection was applied for either injection extracted from herbs (including angelica injection, astragalus injection, and Shenmai injection) or injection of western medicine (including dexamethasone, lidocaine, vitamin B1, vitamin B6, vitamin B12, vitamin K1, vitamin K3, and anticholinergic agent).

Out of the 1,217 reports, 1,193 (98.03%) described the detailed TCM treatment regimens, 1,017 (83.57%) described dosage, and 784 (64.42%) described treatment course. Altogether there were 732 (60.15%) reports described that completed the description of the treatment.

The conventional therapies were mainly chemotherapy, surgery, and radiotherapy. Transcatheter arterial chemotherapy applied in 24 (4.45%) reports was to treat liver cancer and lung cancer mainly.

### 3.5. Outcome Measures Reported

 Symptom relief was the most frequently reported outcome measure in 1,120, (92.03%) reports with 84,380 (90.78%) patients, followed by survival in 410 (33.69%) reports with 48,213 (51.87%) patients, quality of life in 228 (18.73%) reports with 13,143 (14.14%) patients, reducing adverse reactions induced by chemo/radiotherapy in 168 (13.80%) reports with 7,539 (8.11%) patients, relapse in 73 (6.00%) reports with 3,726 (4.01%) patients, metastasis in 53 (4.35%) reports with 2,478 (2.67%) patients, reducing adverse reactions after surgery in 17 (1.40%) reports with 1,079 (1.16%) patients, and safety in 169 (13.89%) reports with 27,230 (29.30%) patients.

Among the 652 reports for treatment of cancer, symptom relief was the most commonly reported outcome in 576 (88.34%) reports with 54,563 (89.40%) patients. Survival was reported in 360 (53.68%) reports with 44,772 (73.35%) patients, in the way of reporting the exact length of survival or rate of survival. In the rest, symptom relief was also the most commonly reported in 544 (96.28%) reports with 29,837 (92.70%) patients. Survival was reported in 51 (9.03%) reports with 3,481 (10.81%) patients.

Totally 228 (18.73%) reports reported quality of life, 153 (67.11%) mentioned the Karnofsky performance status scale, a standardized measurement for quality of life, and 6 (2.63%) mentioned other status scales (ECOG-WHO performance status EORTC QLQ-C30 and self-designed scales) [11]. The rest were considered as unclear reports with typical descriptions: “the patient is in high quality of life now” or “the patient is in a stable condition now” [12].

Out of the 1,217 reports, 414 (34.02%) reported composite outcomes, which described outcomes as predefined grades such as cured, improved, and ineffective, within each grade involved the evaluation of more than one original outcome [13]. All of the 1,217 reports were retrospective studies, in which data were collected basically from medical records and without clear description of follow-up.

### 3.6. Adequacy of Reporting

 Out of the 1,217 reports, 1,182 (97.12%) reported structurally, but not in a structured manner including objective, methods and material (involving participants, treatment regimens, and outcome measures), results, discussion, and/or conclusion. Most of the reports lacked subheadings such as objectives, conclusion, and discussion. 12 (0.99%) reports lacked subheading of result, 95 (7.81%) reports lacked subheading of conclusion, and 37 (3.04%) lacked subheading of discussion.

There were 29 (2.38%) reports that lacked data of patients' baseline information concerning age, sex, and cancer or cancer-related conditions, 7 (0.58%) reports lacked data of treatment regimens concerning dosage, administration, and course. A total of 169 (13.89%) reports provided recommendation for clinical application in conclusion or discussion section, while the rest objectively reported the advantages and disadvantages or did not provide.

## 4. Discussion

### 4.1. Description of Current Situation of TCM for Cancer Care

This review has comprehensively identified and summarized cancer case series for the first time, and 1,217 reports were found in the area of TCM for cancer from the year 1958 to 2011. This brings us an overview and also can provide information for further research and clinical application.

The number of publications per year increased from 1970s with the peak in 2008, which is consistent with findings in a review of case reports in cancer care [2]. During the literature search and study selection, after evaluating full texts most reports were excluded as non-case-series, especially for reporting “control” or “randomized.” This might indicate that researchers have paid more attention to higher-level evidence from 2008. Another possibility is publication lag in different Chinese databases for the year 2009 and 2010, thus an update searching should be conducted in the future to check whether there are more publications identified in the more recent time.

Cancer types treated by TCM therapies in case series covered 10 categories. Either calculated by number of publications or patients, the most frequently reported cancer types were generally consistent with the most common types of cancer (lung, stomach, liver, breast, and esophageal cancer) causing death in Asia and in China [14]. Leukemia, the most frequently reported cancer type in a review of case reports in cancer care [2], was the 4th or 5th most frequently reported cancer type treated by TCM in this review. Leukopenia induced by chemo/radiotherapy was a common type of conditions treated by TCM. These findings might indicate that TCM therapies might have potential advantages for treating hematologic disease.

### 4.2. Recommendations for Structured Reporting

From a methodological point of view, we found the reporting of current TCM case series is in need of improvement. Though almost all the 1,217 reports structurally reported, they were lack of some important sections or contents, or they reported two or more sections in one section. Most case series reported in Chinese journals were reported in structure of information of participants, treatment regimens, results, and discussion, while case series reported in foreign countries were based on clinical trial reporting format [15]. Based on the findings, we recommend that when reporting a TCM case series, the following is necessary.

1. Objectives. All the 1,217 reports were retrospective without a section for objectives, which could show the researchers' clinical questions to be studied. We recommend that all the case series, both prospective and retrospective, should present clear objectives, scientific background, and explanation of rationale in the beginning of the reports.
2. Diagnostic Criteria. A majority of case series reported clinical diagnostic criteria, but just more than half reported gold diagnostic standard and syndrome differentiation based on TCM theory, which should be reported to show the eligibility criteria of patients.
3. Treatment Regimens. Most of the case series did not report the completed information of treatment. Detailed description of ingredients of herbal medicine, regimens, dosage, and course should be reported for clinical application.
4. Outcome Measures. Symptom relief was the most frequently reported outcome in this review. We recommend that the researchers in the future pay more attention to terminal outcomes (survival, relapse, or metastasis). For quality of life (QOL), just more than half of the reports mentioned the Karnofsky performance status scale. We recommend that in future studies the international standardized measurement should be applied.
5. Results. Most of the results reported as composite outcome, which could provide very limited information for clinical application and research. We recommend reporting result according to specific outcome measures.
6. Conclusion and Discussion. Only a small part of the case series reported the authors' recommendation for clinical application. We recommend that the researchers in the future pay more attention to generate suggestions for clinical application and further research.

A recommendation for the full format of the reporting case series is that it should involve abstract, introduction, objectives, material and methods (including participants, treatment regimens, and outcome measures), results, discussion, and/or conclusion.

### 4.3. Potential for Further Studies

Since the reports involved in this review were all retrospective case series, we could just provide an overview of TCM for cancer treatment without any recommendation of specific treatment for clinical application. Therefore, we encourage that higher-level prospective studies (controlled clinical trials or randomized controlled trials) with scientific hypothesis could be established to assess the efficacy and safety of TCM for cancer.

Based on TCM therapies used in case series for cancer, we suggest that further studies can focus on the most frequently applied TCM therapies, Chinese herbal medicine, especially formula based on syndrome differentiation.

Our findings also revealed the most common types of cancer treated by TCM in case series, which indicated the potential benefit of TCM therapies for those types of cancer. Therefore, we recommend that further studies should focus on the most common types of cancer, namely, lung cancer, liver cancer, stomach cancer, leukemia, and esophageal cancer on TCM therapy or integrative therapy.

## Figures and Tables

**Table 1:** Cancer and cancer-related conditions in case series published in Chinese journals.

Name of condition	No. of publications (a)	Frequency (a/1,217)	No. of patients (b)	Frequency (b/92,945)
Cancer	652	53.57%	61,035	65.67%
Subtotal	652	53.57%	61,035	65.67%
Precancerosis	16	1.31%	848	0.91%
Subtotal	16	1.31%	848	0.91%
Pain	92	7.56%	7,275	7.83%
Leukopenia	35	2.88%	1,864	2.01%
Effusion in thoracic or abdominal cavity	38	3.12%	1,363	1.47%
Hiccup	36	2.96%	1,231	1.32%
Inflammation	21	1.73%	1,038	1.12%
Nausea and vomiting	13	1.07%	902	0.97%
Intestinal obstruction	10	0.82%	463	0.50%
Edema	11	0.90%	400	0.43%
Fever	9	0.74%	274	0.29%
Diarrhea	8	0.66%	212	0.23%
Depression	2	0.16%	100	0.11%
Subtotal	275	22.60%	15,122	16.27%
Other adverse reactions or complications	274	22.51%	15,940	17.15%
Subtotal	274	22.51%	15,940	17.15%

Total	1,217	100%	92,945	100%

**Table 2:** Type of cancer treated by TCM in case series published in Chinese journals.

Type of cancer	No. of publications (a)	Frequency (a/652)	No. of patients (b)	Frequency (b/61,035)
Digestive system				
Liver	148	22.70%	5,627	9.21%
Stomach	110	16.87%	3,431	5.62%
Intestine	74	11.35%	2,076	3.40%
Anus/anal rectum	39	5.98%	1,059	1.73%
Esophagus	51	7.82%	3,019	4.94%
Pancreas	28	4.29%	550	0.90%
Gallbladder	4	0.61%	21	0.03%
Subtotal	454	69.62%	15,783	25.84%
Respiratory system				
Lung	158	24.23%	14,298	23.40%
Subtotal	158	24.23%	14,298	23.40%
Hematologic/lymphoid system				
Leukemia	73	11.20%	3,250	5.32%
Malignant lymphoma	16	2.45%	480	0.79%
Subtotal	89	13.65%	3,730	6.11%
Urinary and reproductive systems				
Cervix	24	3.68%	1,457	2.38%
Ovary	17	2.61%	295	0.48%
Bladder	9	1.38%	222	0.36%
Prostate	15	2.30%	217	0.36%
Kidney	5	0.77%	25	0.04%
Penis	1	0.15%	4	0.01%
Subtotal	71	10.89%	2,220	3.63%
Endocrine system				
Breast	60	9.20%	1,677	2.75%
Thyroid	12	1.84%	153	0.25%
Head and neck cancer				
Brain	10	1.53%	92	0.15%
Parotid gland	1	0.15%	2	0.00%
Oral cavity	11	1.69%	83	0.14%
Nasopharynx	32	4.91%	807	1.32%
Larynx	5	0.77%	17	0.03%
Subtotal	59	9.05%	1,001	1.64%
Skeletal and muscular systems	11	1.69%	178	0.29%
Skin cancer	11	1.69%	164	0.27%
Multiple myeloma	7	1.07%	137	0.22%
Malignant mole	4	0.61%	8	0.01%

Total*	652		61,035	

*Some reports involved more than one type of cancer.

**Table 3:** TCM therapies for cancer in case series published in Chinese journals.

TCM therapy	No. of publications (a)	Frequency (a/1217*)	No. of patients (b)	Frequency (b/92945)
Herbal medicine	1102	90.55%	86,741	93.33%
Formula based on syndrome differentiation	703	57.76%	59,454	63.97%
Self-prescribed formula	127	10.44%	26,632	28.65%
Chinese patent medicine	140	11.50%	12,804	13.78%
Preparation used in specific hospital	107	8.79%	7,058	7.59%
Injection extracted from herbs	114	9.37%	5,753	6.19%
Single-herb preparation	30	2.47%	1,234	1.33%
Classical formula or concerted formula	22	1.81%	833	0.90%
Acupoint stimulation	160	13.15%	8,371	9.01%
Manual acupuncture	79	6.49%	3,801	4.09%
Acupuncture point injection	57	4.68%	2,900	3.12%
Electroacupuncture	11	0.90%	1,029	1.11%
Moxibustion	19	1.56%	799	0.86%
Ear acupuncture	12	0.99%	721	0.78%
Cupping	2	0.16%	92	0.10%
Dietary therapy	12	0.99%	667	0.72%
*Qigong* therapy	7	0.58%	2,117	2.28%
Massage (*Tuina*)	6	0.49%	214	0.23%

*Combination of TCM therapies was described in some reports.
